# Tumor-associated myeloid cells promote tumorigenesis of non-tumorigenic human and murine prostatic epithelial cell lines

**DOI:** 10.1007/s00262-018-2143-y

**Published:** 2018-03-03

**Authors:** Stephanie N. Sass, Kimberley D. Ramsey, Shawn M. Egan, Jianmin Wang, Eduardo Cortes Gomez, Sandra O. Gollnick

**Affiliations:** 1 Department of Immunology, Roswell Park Cancer Institute, Elm and Carlton Sts, Buffalo, NY 14263 USA; 2 Department of Cell Stress Biology, Roswell Park Cancer Institute, Elm and Carlton Sts, Buffalo, NY 14263 USA; 3 Department of Biostatistics and Bioinformatics, Roswell Park Cancer Institute, Buffalo, NY USA

**Keywords:** Myeloid cells, Prostate tumorigenesis, IL-1α, CXCL8, Angiogenesis

## Abstract

The etiology of prostate cancer is poorly understood, but it is a multi-step process that has been linked to environmental factors that induce inflammation within the gland. Glands of prostate cancer patients frequently contain multiple zones of disease at various stages of progression. The factors that drive disease progression from an indolent benign stage to aggressive disease are not well-defined. Prostate inflammation and carcinoma are associated with high levels of myeloid cell infiltration; these cells are linked to disease progression in other cancers, but their role in prostate cancer is unclear. To determine whether myeloid cells contribute to prostate cancer progression, the ability of prostate tumor-associated CD11b^+^ cells (TAMC) to drive prostate epithelial cell tumorigenesis was tested. Co-culture of CD11b^+^ TAMC with non-tumorigenic genetically primed prostate epithelial cells resulted in stable transformation and induction of tumorigenesis. RNA sequencing identified the IL-1α pathway as a potential molecular mechanism responsible for tumor promotion by TAMC. Inhibition of IL-1α delayed growth of TAMC-induced tumors. Further analysis showed that IL-1α inhibition led to decreased angiogenesis within tumors, suggesting that IL-1α promotes prostate tumor progression, potentially through augmentation of angiogenesis.

**Electronic supplementary material:**

The online version of this article (10.1007/s00262-018-2143-y) contains supplementary material, which is available to authorized users.

## Introduction

Prostate cancer is the most common non-cutaneous malignancy and second leading cause of death in American men. The disease develops slowly and is associated with age. The glands of older men frequently contain multiple areas of abnormalities, which have been postulated to represent a “field of cancerization” consisting of activated pre-malignant lesions, including proliferative inflammatory atrophy (PIA) and prostatic intraepithelial neoplasia (PIN) and small carcinomas, [1, 2]. Although evidence of progression of these lesions to prostate cancer is limited [1], study of pre-malignant conditions has shown that they can provide a setting for transformation to progressive disease [1, 3]. Several studies have suggested that chronic inflammation of the prostate contributes to disease progression [1, 4].

Inflammation of the prostate is associated with myeloid cell infiltrate that increases with disease progression [5]. The role of these cells in the transition from indolent to progressive disease is unclear. In this communication, we examined whether myeloid cells isolated from prostate tumors could induce tumorigenesis in non-tumorigenic, genetically primed prostate epithelial cells. These cells model non-tumorigenic cells found in human prostate glands that have undergone genetic assault over time and are primed to develop into progressive disease following further genetic alteration. We demonstrate that tumor-associated myeloid cells isolated from either murine tumors or human tumor xenografts are capable of driving stable tumorigenesis in prostatic epithelial cell lines. Myeloid-driven tumorigenic cells exhibited enhanced activation of the IL-1α pathway as determined by RNA sequencing. The IL-1α pathway target CXCL8, which is associated with prostate cancer disease progression and reduced survival [6, 7], was up-regulated in myeloid-driven tumor cells. Both in vitro and in vivo blockade of the IL-1α pathway with its natural antagonist IL-1Ra [8] abolished expression of downstream targets of IL-1α and significantly decreased prostate tumor growth and angiogenesis. Reduction of IL-1α expression by prostate tumor cells with shRNA (short hairpin RNA) also significantly inhibited tumor growth. These findings suggest that IL-1α plays a critical role in the transition from indolent to progressing prostate cancer and that IL-1α may serve as a future prognostic indicator for diagnosing progressing disease.

## Materials and methods

### Materials

PCR and qPCR primers were purchased from IDT (Coralville, IA). Antibodies specific for CD45 (552950, clone 104), CD11b (557657, clone M1/70), Ly6G (551460, clone 1A8), Ly6C (561237, clone AL-21), CD11c (550261, clone HL3) and all isotypes were purchased from BD Pharmingen (Mountain View, CA, USA). The antibody specific for F4/80 (25-4801-82, clone BM8) and its isotype were purchased from eBioscience (San Diego, CA, USA). Recombinant human and mouse IL-1α and recombinant human IL-1Ra were purchased from Peprotech (Rocky Hill, NJ, USA). The doxycycline-inducible pQCXIX RT3GEPIR construct was kindly provided by Dr. S. Olejniczak (RPCI) [9].

### Cell lines

The human prostate cancer cell line, PC-3M, was obtained in Spring 2010 from Dr. I. Gelman (RPCI) and cultured in 10% FBS at 37 °C and 5% CO_2_. The nonneoplastic immortalized prostate epithelial BPH-1 cells were kindly provided by Dr. B. Foster (RPCI) in Spring 2010 and cultured in 10% FBS at 37 °C and 5% CO_2_. All cells were examined for mycoplasma contamination in February 2015. E6 shIL-1α and scrIL-1α cells were generated by retroviral transduction; these constructs are doxycycline-inducible and the cells were propagated under puromycin selection (1 µg/ml). The tumorigenic TRAMP C2 and non-tumorigenic TRAMP C3 murine prostate cancer cell lines were kindly provided by Dr. B. Foster (RPCI) in Spring 2010 and maintained as described in the presence of 10^−8^ M dihydrotestosterone (Sigma) at 37 °C and 10% CO_2_ [10].

### Animals

Male severe combined immunodeficiency (SCID) pathogen-free mice aged 7–8 weeks were purchased through the Laboratory Animal Resource of RPCI. Male C57BL/6 pathogen-free mice aged 6–8 weeks were purchased from Taconic (Hudson, NY, USA). Animals were housed in individually ventilated microisolator cages in a limited access barrier facility in laminar flow units under ambient fluorescent light.

### In vivo tumor growth and IL-1Ra studies

Tumor cells and cells of admixtures were counted and had their viability confirmed to be greater than 95% prior to use in vivo. Male SCID mice were injected subcutaneously in the flank with 10^6^ cells (BPH-1, BPH-TW or PC-3M). Male C57BL/6 mice were injected subcutaneously in the flank with 10^6^ TRAMP C2, TRAMP C3 or C3X cells. In the admix experiments, TRAMP C3 cells were mixed with MACS-purified, MHC-matched CD11b^+^ cells at a 2:1 ratio with 10^6^ cells total, respectively, prior to subcutaneous implantation. Tumor growth was measured with calipers in two dimensions; tumor volume, *V*, was calculated using the formula *V* = (*lw*^2^/2), where *l* is the longest axis of the tumor and *w* is the axis perpendicular to *l*. For the IL-1Ra studies, SCID mice were injected subcutaneously with 5 × 10^5^ E6 cells, with or without 2.5 µg IL-1Ra in the tumor cell suspension. Mice were subsequently injected intraperitoneally every 3 days with 2.5 µg IL-1Ra (Peptrotech) or PBS for the duration of the experiment. Mice injected with either 10^6^ E6 shIL-1α or E6 scrIL-1α cells were given doxycycline-containing chow (2018, 625 doxycycline from Harlan Labs) for the entirety of the experiment.

### Isolation of myeloid cells

Tumor or splenic CD11b^+^ cells were purified from PC-3M or C2 tumor-bearing mice. Selections were performed using CD11b^+^ magnetic beads from Miltenyi Biotec (Auburn, CA, USA) on LS MACS columns. The purity of the enriched CD11b^+^ cells was determined by flow cytometry following each separation and the percentage of CD11b^+^ cells was routinely > 95%.

### Flow cytometry

Single cell preparations of tumors by enzymatic digestion or mechanically digested spleens were pre-incubated with normal mouse IgG (Invitrogen, Grand Island, NY, USA) to block Fc receptor binding, followed by incubation with directly conjugated primary monoclonal antibodies. Labeled cells were collected on an LSRFortessa (BD Biosciences, San Jose, CA, USA) and analyzed by WinList software. Definition of myeloid cell populations found in Supplementary Table 1.

### Long-term transwell assays

Transwell plates (10 cm; 0.4 µm pore size) were used for co-culture experiments (Corning Incorporated, Corning, NY, USA). CD11b^+^ cells were isolated from PC-3M tumors or spleens of tumor-bearing mice (see above) and plated in the top well at a concentration of 2.5 × 10^5^ cells. BPH-1 cells were plated in the bottom of the transwell at a concentration of 5 × 10^5^ cells. The cells were co-cultured at 37 °C and 5% CO_2_ for 5 days after which the BPH-1 cells were harvested, counted and viability was determined prior to injection of 10^6^ cells subcutaneously into the flanks of SCID mice. Protocol adapted from [11].

### Genomic DNA (gDNA) extraction and RT-PCR

Genomic DNA was harvested from BPH-1 and BPH-TW cells by phenol chloroform extraction. The Platinum Taq DNA Polymerase kit (Invitrogen) was used and PCR reactions were run on a Bio-Rad C1000 Thermal Cycler. PCR products were run on a 1.5% agarose gel and the gel was imaged on a Bio-Rad ChemiDoc XRS + System.

### RNA extraction and quantitative Real-Time PCR

Total RNA was extracted from cells using Trizol^®^ reagent (Invitrogen) according to manufacturer’s directions. Real-time quantitative PCR was performed using the relative standard curve method to analyze target gene expression. cDNA was synthesized using the iScript cDNA Synthesis Kit (Bio-Rad). qRT-PCR was performed in the CFX96 Real-Time PCR Detection System (Bio-Rad) in a 20 µl reaction volume using the SsoAdvanced Universal SYBR Green Supermix (Bio-Rad), according to the manufacturer’s recommendations. Fluorescence was measured following each cycle and analyzed. Specificity of PCR products was confirmed by melting curve analyses. For each sample analyzed, the mean 2^−∆*Ct*^ value based on the results of all experiments was calculated, together with that of the corresponding standard samples. Relative amount of gene mRNAs were normalized for loading differences by GAPDH gene mRNA. All samples were treated in duplicate and experiments were repeated in triplicate. Results are reported as relative mRNA expression. qRT-PCR primer sequences found in Supplementary Table 2.

### RNA sequencing

Total RNA from BPH-1, E6, E4 and G6 cells was extracted using the Aurum Total RNA Mini Kit (Bio-Rad) and quantified by a SmartSpec Plus spectrophotometer (Bio-Rad). Sample requirements were as follows: RIN > 7 and an OD 260:280 of 1.9–2.0. RNA samples were given to the Genomics Core at RPCI where the quality control, RNA sequencing, and analysis was performed.

### Cytokine analysis

Cell-free supernatants from tumor cell lines were collected after incubation for various time points with or without IL-1Ra treatment. Cell-free supernatants were also collected from E6 shIL-1α and scrIL-1α cell lines after treatment with doxycycline throughout a time course. All samples were stored at − 20 °C until assayed. ELISA kits specific for human IL-1α were purchased from R&D systems. Assays were completed according to manufacturers’ instructions.

### Immunohistochemistry

Immunohistochemical analyses of human xenograft tissue in mice included staining tissues with an antibody specific for CD31. Briefly, E6 tumors (~ 400 mm^3^) were harvested from mice that had received either IL-1Ra or PBS injections, fixed in zinc formalin (BD Pharmingen) and paraffin embedded. Slides were stained by the Pathology Network Facility at RPCI with hematoxylin and eosin and stained for CD31.

### Statistical analysis

Statistical analyses were performed using a standardized Student *t* test with Welch’s correction, where equal variances were not assumed, to compare experimental groups. Differences were considered significant when *P* values were ≤ 0.05.

## Results

### Conversion of non-tumorigenic prostate epithelial cells to tumorigenic cells by tumor-associated myeloid cells

Myeloid cells have been implicated in prostate cancer initiation and progression [5]. However, their role in conversion of benign or indolent disease to progressing disease is not well-understood. To study the link between myeloid cells and prostate cancer progression, we examined the ability of myeloid cells isolated from human PC-3M prostate tumor xenografts in SCID mice to induce tumorigenesis in genetically initiated non-tumorigenic BPH-1 cells. The BPH-1 cell line is derived from human prostate epithelial cells that have been immortalized with SV40 large T antigen [12, 13]. Transformation with SV40 alone does not induce tumorigenesis, but primes these cells for further genetic alteration [12].

**Fig. 1:**
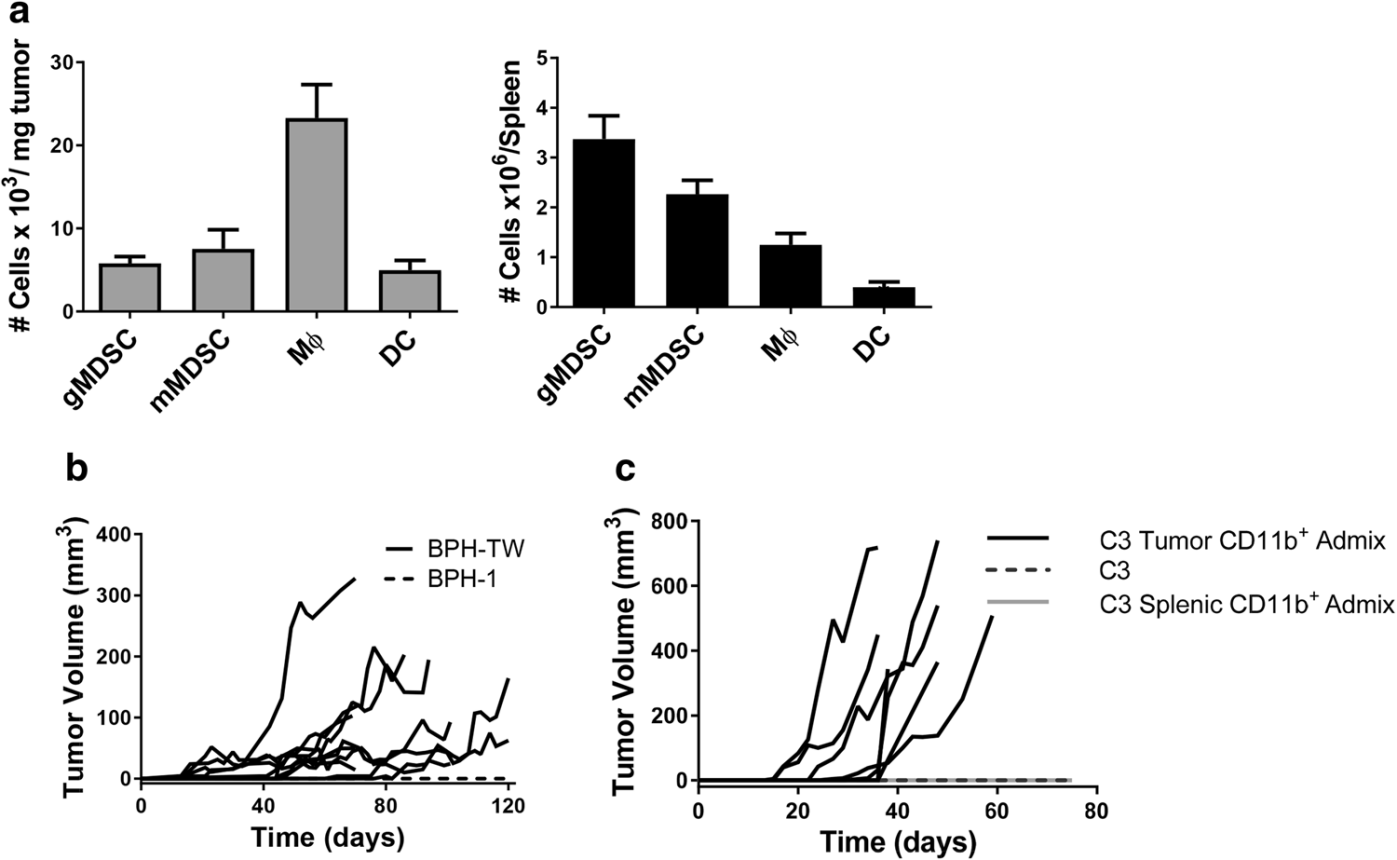
CD11b^+^ tumor-associated myeloid cells convert indolent prostate disease to progressing disease. **a** Flow cytometric analysis of myeloid cell infiltrate in PC-3M tumors and spleens of tumor-bearing mice. Results are presented as the mean of four experiments ± SEM. **b** Tumor growth kinetics in SCID mice injected with either 10^6^ BPH-1 cells from the CD11b^+^ co-culture (*n* = 10) or BPH-1 cells cultured alone (*n* = 5). Each line represents tumor growth in an individual mouse. **c** Tumor growth kinetics in C57BL/6 mice injected with TRAMP C3 (C3) cells (*n* = 5) or admixtures of C3 cells and either tumor-derived CD11b^+^ myeloid cells (*n* = 7) or splenic-derived CD11b^+^ myeloid cells (*n* = 5). Each line represents tumor growth in an individual mouse

PC-3M tumor-associated myeloid cells are a heterogeneous population, with the dominant subset consisting of F4/80^+^ macrophages (Fig. 1a). Ly6G^lo/−^Ly6C^hi^ M-MDSC (monocytic phenotype), Ly6G^hi^Ly6C^lo/−^ PMN-MDSC (polymorphonuclear phenotype) and CD11c^+^ dendritic cell (DC) populations were also present, although in lower numbers. In contrast, PMN-MDSC were the most abundant myeloid subset in the spleens of mice bearing PC-3M tumors, while the F4/80^+^ macrophages and CD11c^+^ DC were much less prevalent (Fig. 1a).

BPH-1 cells were co-cultured in transwell dishes with CD11b^+^ myeloid cells isolated from PC-3M tumors for 5 days. On day 5 of co-culture, the BPH-1 cells were harvested and injected subcutaneously into male SCID mice; tumor growth was monitored for 120 days or until tumors reached 400 mm^3^. Tumor establishment and growth was observed in nine out of ten mice, although tumors grew at varying rates (Fig. 1b). Histologic examination of the myeloid cell-driven BPH-1 tumors indicated that they exhibit a squamous phenotype (data not shown).

The ability of tumor-associated myeloid cells to promote tumorigenicity was confirmed using cell lines derived from the autochthonous TRAMP murine model of prostate cancer, which expresses SV40 large and small T antigen under control of the probasin promoter. The isogenic cell lines TRAMP C2 and C3 were derived from a single tumor and display different tumorigenic potential [10]. C2 cells are highly tumorigenic in C57BL/6 mice, while TRAMP C3 cells are non-tumorigenic in either C57BL/6 or SCID mice. The myeloid cell infiltrate of C2 tumors is similar in subpopulation composition to that of PC-3M tumors (Supplementary Fig. 1). TRAMP C3 tumor cells were admixed with myeloid cells isolated from C2 tumors and injected subcutaneously into immune-competent C57BL/6 mice. Tumors grew in all mice injected with the admixture; admixture with splenic CD11b^+^ myeloid cells did not promote tumorigenesis (Fig. 1c).

### Tumor-derived myeloid cell induced tumorigenicity is stable

**Fig. 2:**
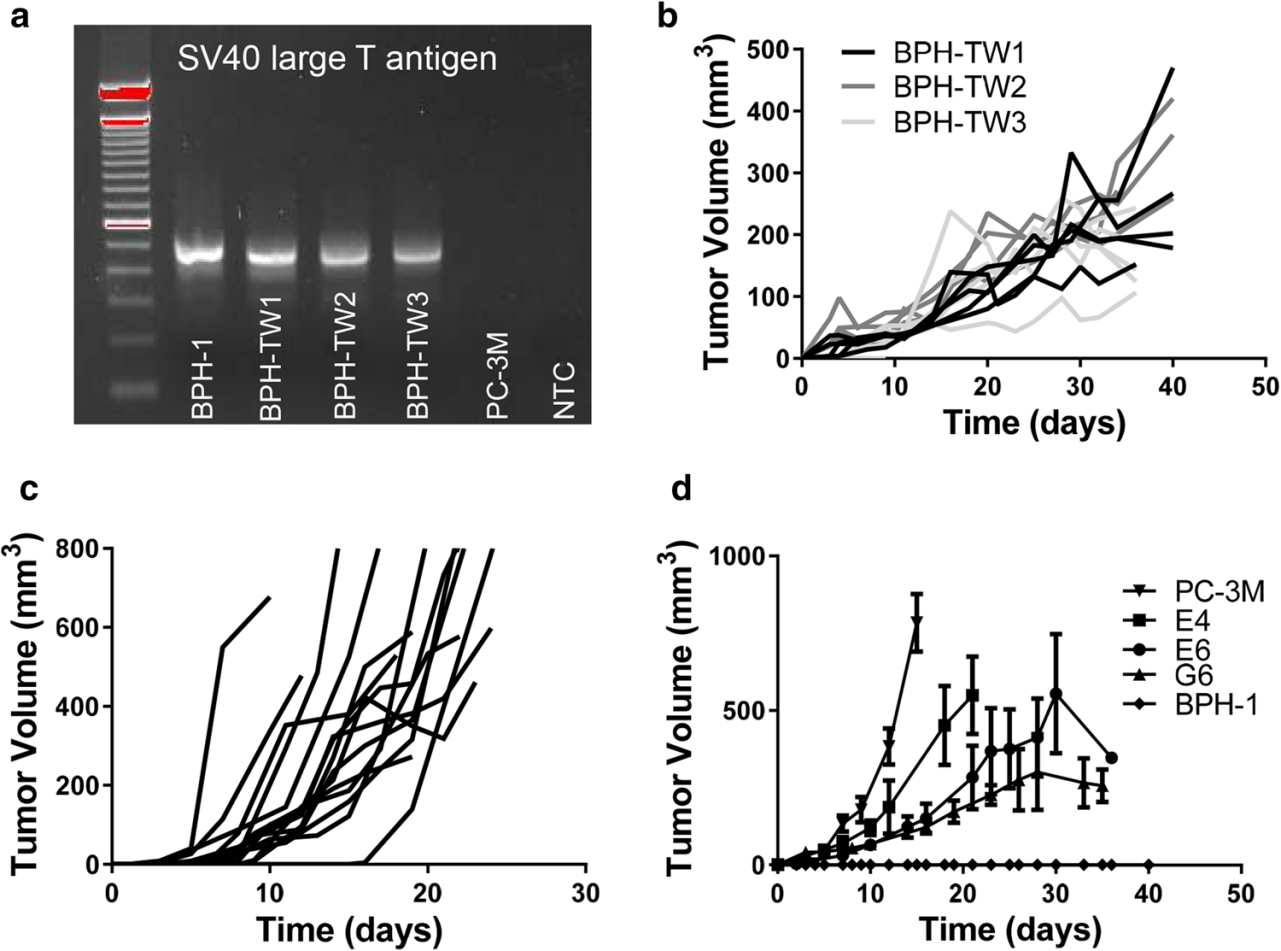
Tumor-derived myeloid cell induced tumorigenicity is stable over time. **a** Presence of SV40 large T antigen gDNA in parental BPH-1 cells, BPH-TW1-3 and PC-3M cells. **b** Tumor growth kinetics in SCID mice injected with BPH-TW cell lines. Each line represents tumor growth in an individual mouse (BPH-TW1 *n* = 6, BPH-TW2 *n* = 4, BPH-TW3 *n* = 4). **c** Tumor growth kinetics in C57BL/6 mice injected with cells derived from tumors arising following injection of C3 cells with C2-derived myeloid cells. Each line represents tumor growth in an individual mouse. **d** Tumor growth following injection of SCID mice with BPH-TW single cell clones, PC-3M, and BPH-1 (*n* = 4 per group)

Single cell suspensions were generated from three myeloid cell-driven BPH-1 tumors and expanded in culture; the resultant cell lines were termed BPH-TW (BPH-transwell). The origin of BPH-TW cells was confirmed by SV40 large T antigen expression (Fig. 2a). BPH-TW cell lines also exhibited similar cobblestone morphology to that of BPH-1 cells and have undergone epithelial to mesenchymal transition (EMT; Supplementary Fig. 2). BPH-TW cells were injected into naïve SCID mice in the absence of additional myeloid cells and tumor growth was monitored. BPH-TW cell lines remained tumorigenic (Fig. 2b). Similarly, tumorigenicity of cells derived from myeloid-driven C3 tumors (C3X) was stable following expansion in vitro (Fig. 2c).

### RNA sequencing implicates the IL-1 pathway in conversion of indolent disease to progressing disease

**Fig. 3:**
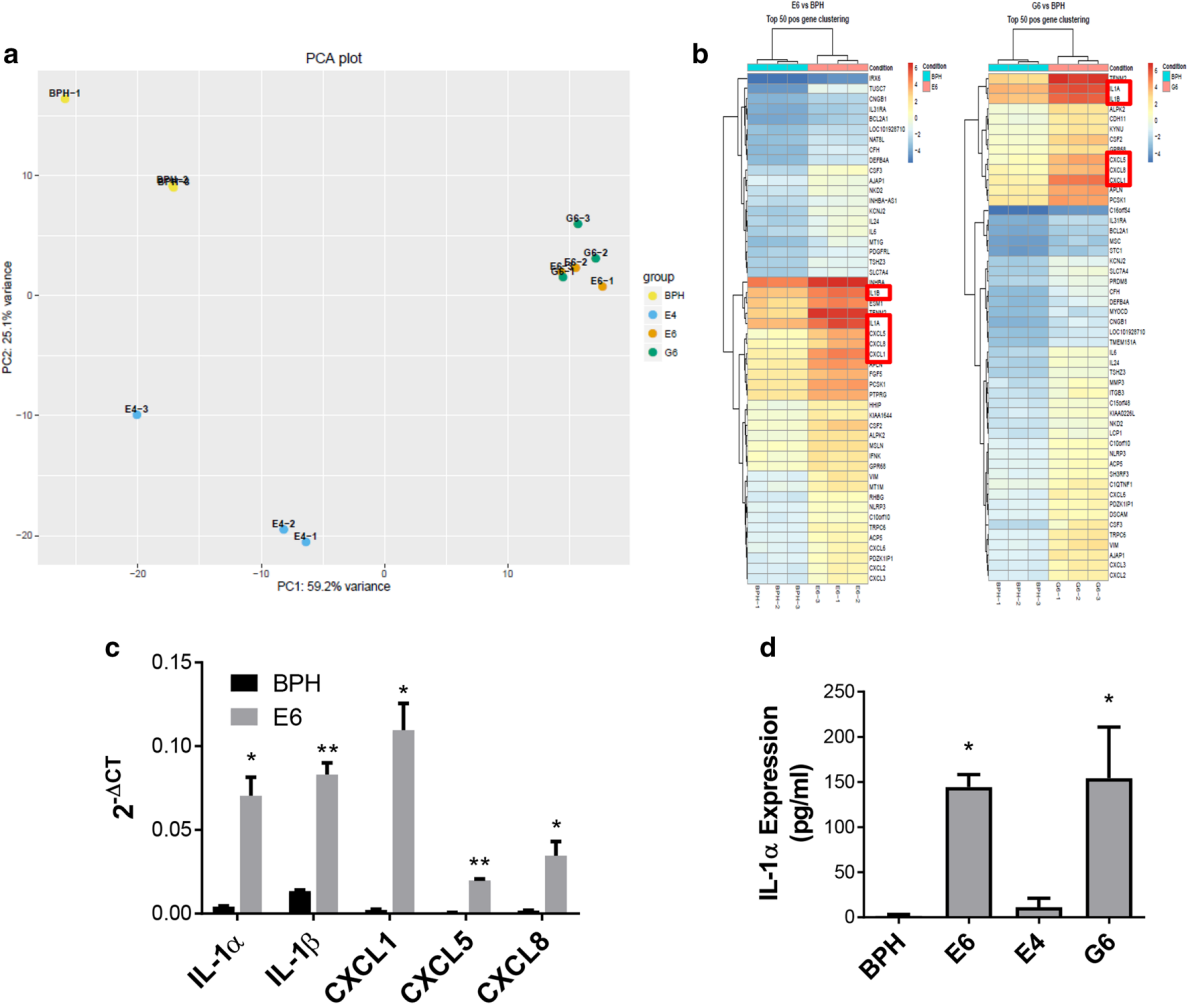
RNA sequencing implicates the IL-1 pathway in conversion of indolent disease to progressing disease. **a** The PCA plot. **b** Clustergrams comparing parental non-tumorigenic BPH-1 RNA to tumorigenic BPH-TW clones E6 and G6. Red boxes indicate IL-1 pathway target genes. **c** qRT-PCR of IL-1 pathway genes. Results are presented as the mean of three experiments ± SEM; *P < 0.02, **P < 0.001 when compared to BPH-1 expression. **d** IL-1α protein expression levels were examined by ELISA. Results are presented as the mean of three experiments ± SEM (CXCL8 mean of four experiments); *P < 0.02 when compared to IL-1α secretion by BPH-1 parental cells. Significance was evaluated using Student *t* test

To further understand the changes induced by myeloid cell conversion of BPH-1 cells, single cell clones from BPH-TW cell lines were generated (Fig. 2d; Supplementary Fig. 3a–c); all clones exhibited rapid and consistent tumor growth. Three clones were randomly selected for subsequent analysis: BPH-TW1 clone E6, BPH-TW2 clone E4 and BPH-TW3 clone G6. RNA sequence analysis showed that gene expression in E6 and G6 cells clustered together (Fig. 3a). Five genes were notably up-regulated in the clustergrams of the tumorigenic E6 and G6 cells compared to the non-tumorigenic BPH-1 cells and the tumorigenic E4 cells: *IL-1α, IL-1β, CXCL1, CXCL5* and *CXCL8* (Fig. 3b). Pathway analysis of the up-regulated genes showed an increase in IL-1 pathway activity in E6 and G6 cell lines; the five genes noted above are all members of this pathway. The IL-1 pathway did not appear to be activated in E4 cells and none of the identified pathway genes were up-regulated in the E4 cells (Supplementary Fig. 4a). qRT-PCR was performed to confirm the increased expression of these genes; levels of IL-1α, IL-1β, CXCL1, CXCL5 and CXCL8 mRNA were significantly higher in the E6 cells compared to BPH-1 (Fig. 3c). Up-regulation of CXCL1 and CXCL8 mRNA was also seen in other single cell clones isolated from BPH-TW1 and BPH-TW3, but not in single cell clones isolated from BPH-TW2 (Supplementary Fig. 4 b, c). Parental BPH-1, BPH-TW1 clone E6, BPH-TW2 clone E4 and BPH-TW3 clone G6 were also tested for protein expression of IL-1α; both E6 and G6 cells secreted IL-1α, while E4 and BPH-1 cells did not (Fig. 3d).

### IL-1α contributes to myeloid cell induced tumorigenesis

**Fig. 4:**
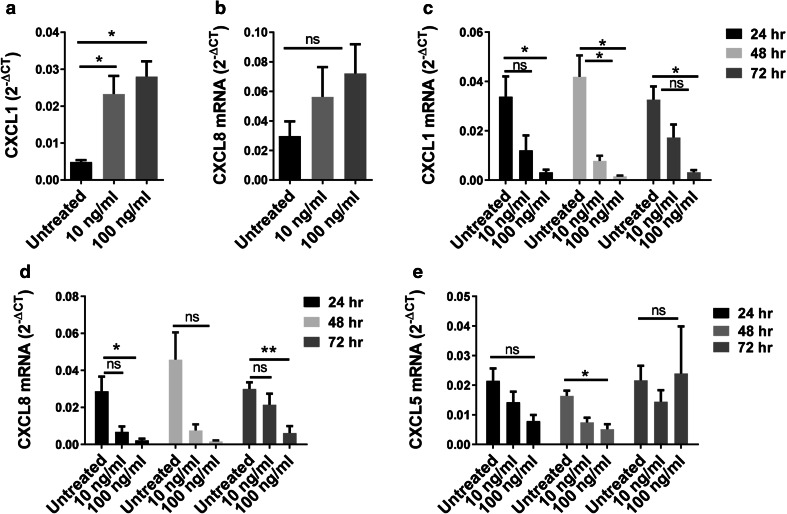
In vitro IL-1α modulation alters expression of IL-1α target genes. **a, b** Expression of CXCL1 and CXCL8 by BPH-1 cells following 5 day stimulation with recombinant human IL-1α. Results are presented as the mean of four experiments ± SEM; *P < 0.03 when compared to expression in untreated cells. **c**–**e** Expression of CXCL1, CXCL5 and CXCL8 by E6 cells following stimulation with recombinant human IL-1α. Results are presented as the mean of four experiments ± SEM; *P < 0. 03, **P < 0.006 when compared to expression in untreated cells

The IL-1α pathway has been implicated in a feed-forward signaling loop that leads to the production of pro-tumorigenic factors that contribute to malignant transformation, tumor formation and production of angiogenic factors, including CXCL1 and CXCL8 [14–16]. Elevated CXCL8 has been linked to worse overall survival in prostate cancer [7, 17]. Activation of the IL-1α pathway in BPH-1 cells resulted in increased expression of CXCL1 and CXCL8 (Fig. 4a, b), as well as CXCL5 and IL-1α (Supplementary Fig. 5a, b). Inhibition of the IL-1α pathway in E6 cells by IL-1Ra, a natural antagonist of the IL-1 receptor, resulted in a significant decrease in CXCL1 and CXCL8 expression (Fig. 4c, d), but had nominal effect on CXCL5 (Fig. 4e).

**Fig. 5:**
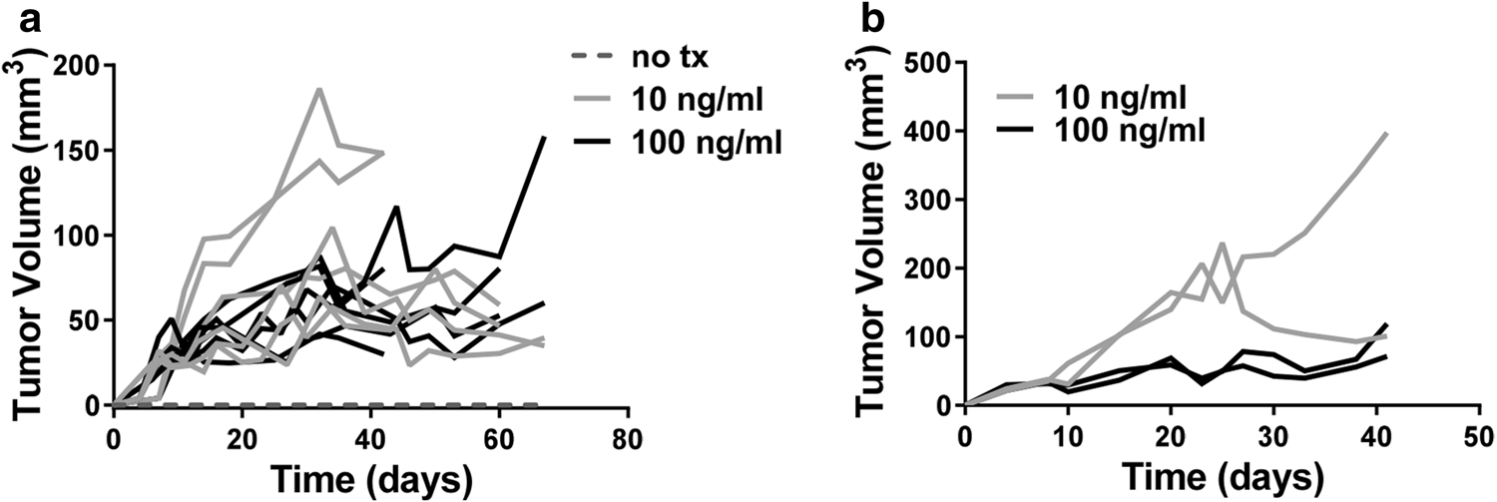
In vivo IL-1α pathway modulation alters tumor growth by BPH-1 cells. **a** Tumor growth kinetics in SCID mice injected with BPH-1 cells treated for 5 days with recombinant human IL-1α. Each line represents tumor growth in an individual mouse. **b** Tumor growth kinetics in SCID mice injected BPH^rhIL1α^ cells. Each line represents tumor growth in an individual mouse

To determine whether activation of the IL-1α pathway was sufficient to induce tumorigenicity in genetically primed prostate epithelial cells, BPH-1 cells were treated for 5 days with increasing doses of rhIL-1α. IL-1α activated and untreated BPH-1 cells were injected into SCID mice and tumor growth was monitored. Incubation with both 10 and 100 ng/ml rhIL-1α resulted in tumor establishment and subsequent tumor growth (Fig. 5a). IL-1α-driven BPH-1tumors were explanted and single cell suspensions were generated; the resultant cell lines were termed BPH^rhIL−1α^. BPH^rhIL−1α^ cells were injected into naïve SCID mice and tumor growth was observed (Fig. 5b), suggesting that the initial 5 day treatment with rhIL-1α was enough to promote stable BPH-1 tumorigenesis.

**Fig. 6:**
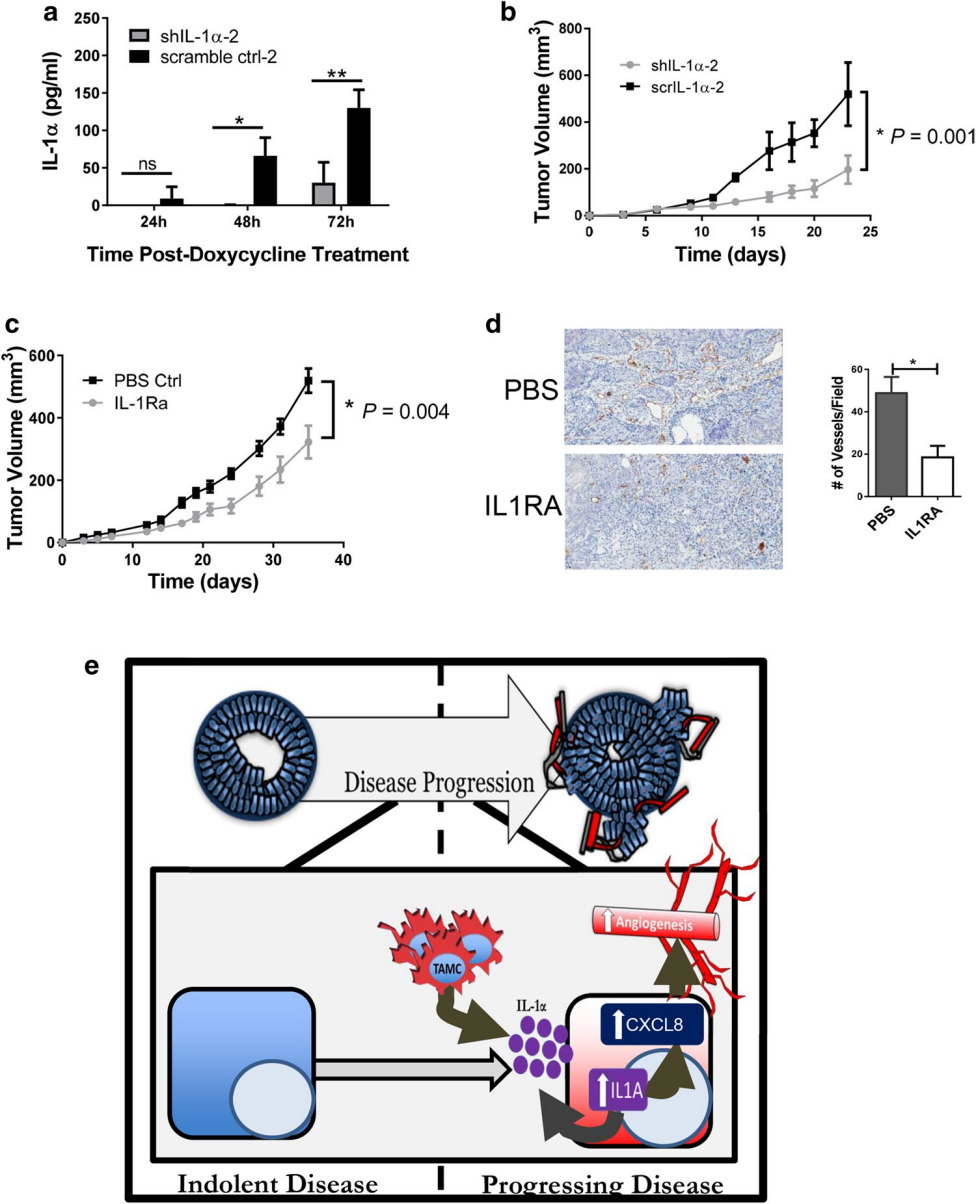
In vivo IL-1α pathway modulation alters tumor growth in E6 cells. **a** IL-1α expression in doxycycline treated E6 shIL-1α and E6 scrIL-1α cells Results are presented as the mean of three experiments ± SEM; *P < 0.04, **P < 0.009. **b** Tumor growth kinetics in doxycycline treated SCID mice injected with E6 shIL-1α or E6 scrIL-1α cells (*n* = *4* per group); **P = 0.001. **c** Tumor growth kinetics in SCID mice injected with E6 cells suspended in either PBS or 2.5 µg IL-1Ra (*n* = 6 per group). IL-1Ra treatment was repeated every 72 h for the length of the experiment; *P = 0.004. **d** Immunohistochemistry for CD31. Results are presented as the mean of three experiments ± SEM; *P < 0.05. **e** Proposed overall model. Tumor-associated CD11b^+^ cells play a role in facilitating the transition from indolent to progressing prostate cancer. Increased vimentin expression in the tumorigenic cells is indicative of EMT. IL-1α expressed by the tumorigenic cells engages IL-1R and induces CXCL8. Expression of CXCL8 facilitates angiogenesis and tumor progression

Activation of the IL-1 pathway in prostate epithelial cells may be affecting the prostate epithelial cells themselves as well as the surrounding microenvironment by stimulating the release of CXCL1 and CXCL8, which are known to enhance angiogenesis. To determine whether IL-1α expression by tumor cells directly affected tumor cell proliferation, two pairs of E6 shIL-1α and E6 scrIL-1α cell lines were generated with doxycycline-inducible retroviral constructs. Treatment of the transduced cells with doxycycline resulted in a 70% (E6 shIL-1α-1 cells) to 90% (E6 shIL-1α-2 cells) decrease in IL-1α expression compared to the corresponding control cells (Fig. 6a; Supplementary Fig. 6a). Reduction of IL-1α expression did not affect tumor cell proliferation in vitro (Supplementary Fig. 6b, c). However, tumor growth was significantly delayed in mice bearing tumors expressing shIL-1α as compared to mice bearing tumors expressing scrIL-1α, supporting a paracrine role for IL-1α in prostate tumorigenesis (Fig. 6b; Supplementary Fig. 6d).

The paracrine effect of the IL-1 pathway on prostate tumor growth was confirmed using IL-1Ra to block the pathway. A significant delay in growth was observed in the mice that received IL-1Ra during tumor growth (Fig. 6c). In contrast, growth of BPH-TW2 clone E4, which does not express elevated levels of IL-1 pathway genes (Supplementary Fig. 4a), was not effected by administration of IL-1Ra (Supplementary Fig. 6e). The delay in tumor growth was accompanied by a reduction in angiogenesis as measured by determining the number of blood vessels present in IL-1Ra treated and control tumors (Fig. 6d), suggesting that IL-1α pathway enhancement of tumor growth may due to increased angiogenesis.

## Discussion

The results reported here demonstrate the ability of tumor-associated CD11b^+^ myeloid cells to convert genetically primed human (BPH-1) and murine (TRAMP C3) prostate epithelial cells from a non-tumorigenic to tumorigenic state. RNA sequence analysis of myeloid-driven tumorigenic BPH-TW cells revealed increased expression of IL-1α and its target genes *CXCL1* and *CXCL8*. shRNA knockdown of IL-1α in the transformed BPH-TW tumor cells resulted in decreased tumor growth.

The IL-1α pathway target CXCL8 (IL-8) is associated with disease progression and overall survival in human prostate cancer. CXCL8 is a pro-angiogenic chemokine; silencing of CXCL8 expression in prostate tumor cells leads to inhibition of angiogenesis and decreased prostate tumor growth [6, 18, 19]. Our studies demonstrate that activation of the IL-1α pathway increases expression of CXCL8 in myeloid-driven BPH-1 tumor cells and that inhibition of the pathway leads to decreased angiogenesis and reduced tumor growth, suggesting that myeloid cells are able to activate the IL-1α pathway in genetically primed prostate epithelial cells, resulting in expression of CXCL8 and increased angiogenesis, which contributes to malignant transformation and tumor growth (Fig. 6e).

The non-tumorigenic models used in this study, BPH-1 and TRAMP C3, are genetically initiated by having been immortalized by expression of SV40 Large T antigen or induced by expression of SV40 Large and small T antigen, respectively [10, 12]. SV40 T inactivates the tumor suppressor pathways mediated by p53 and Rb [20]; alterations in the genes encoding Rb and p53 are associated with prostate cancer progression in patients [21, 22]. However, both BPH-1 and TRAMP C3 cell lines are consistently non-tumorigenic [10, 12, 13, 23]. Prostate cancer is a slow growing malignancy that occurs as a result of a multi-step process, which appears to involve both genetic changes within prostate epithelium and epigenetic changes induced by the surrounding microenvironment [1, 3]. We believe and others have postulated [13] that the cell models used in the current study represent prostate epithelial cells that have undergone a significant genetic assault that has rendered them susceptible to further genetic and epigenetic modifications, such as those promoted by co-culture with tumor-associated myeloid cells, which result in malignant transformation and disease progression.

Prior studies have shown that reactive and tumor-derived stroma can drive tumorigenesis in non-tumorigenic prostate epithelial cells lines [3, 23, 24]. BPH-1 cells can also be driven to a tumorigenic phenotype with hormonal carcinogens; culture of BPH-1 cells with hormonal carcinogens, tumor-derived stroma or tumor-derived myeloid cells used in this study resulted in tumors that are primarily squamous cell carcinomas [13, 23, 24]. Human prostate cancer is primarily adenocarcinoma and squamous prostate cancer is rare. The tendency toward squamous cell differentiation in the BPH-1 model is a limitation of this model system. However, the clear link between inflammation, CXCL8 and myeloid cells with disease progression in humans suggests that our findings have clinical relevancy [4].

The ability of myeloid cells to transform prostate epithelial cells to tumor cells is supported by studies of Fang et al. [11] who showed that co-culture of an acute monocytic cell line (THP-1) with a prostatic epithelial cell line (RWPE-1) could promote tumorigenesis. BPH-1 cells can also be transformed by co-culture with cancer-associated fibroblasts (CAFs) [13]. Incubation with CAFs or THP-1 cells led to EMT of non-tumorigenic prostate epithelial cells in both of these studies. The conversion of BPH-1 cells to BPH-TW cells in our study was also accompanied by EMT. The mechanism by which CAFs convert BPH-1 cells to tumor-forming cells is unclear. Transformation of RWPE-1 by THP-1 depended upon increased CCL4/STAT3 activation, which was not observed in our study. Thus, it appears that conversion of prostate epithelial cells to tumor-forming cells can be mediated by multiple cell types within the stroma and by multiple mechanisms.

The hypothesis that multiple transformation mechanisms can occur following incubation of BPH-1 cells with stromal cells is supported by our results showing differences in BPH-TW cell transcriptomes. Although IL-1α is a critical factor in the transformation of two of the BPH-TW cell lines, the third line, BPH-TW E4, displays a different transcriptome, suggesting an alternative method of transformation. E4 cells appear to have undergone EMT but do not express elevated levels of IL-1α or its target genes; E4 tumor growth is not effected by inhibition of the IL-1 pathway. Thus, a distinct mechanism of transformation has led to tumorigenesis in the E4 cell line. Interestingly, this cell line also did not show increased expression or activation of CCL4 or STAT3, as seen in the Fang study [11].

The mechanism by which myeloid cells are able to induce stable transformation of genetically initiated prostate epithelial cells is unknown. A recent study by Wang et al. demonstrated that tumor-associated macrophages can induce epigenetic changes in gastric cancer cells [25]. Hayward et al. have suggested that cancer-associated fibroblasts induce epigenetic changes in BPH-1 cells [13]. Our results indicate that myeloid-driven BPH-TW cells display a stable transcriptome that is distinct from that of parental BPH-1 cells, suggesting that epigenetic changes have occurred in the transformed cells. The IL-1α gene is susceptible to epigenetic regulation [26]; however, whether tumor-derived myeloid cells have epigenetically altered BPH-1 or TRAMP C3 cells remains to be determined.

The suggestion that the IL-1α pathway contributes to prostate tumorigenesis provides the possibility of novel therapies. Anakinra (brand name Kineret) is a recombinant version of IL-1Ra that is clinically used to treat rheumatoid arthritis, but may have a broader clinical impact in treating solid tumors that have increased IL-1α expression. MABp1, an anti-IL-1α human monoclonal antibody, has recently been tested in treating solid tumors and has shown moderate therapeutic efficacy [27, 28]. Targeted inhibition of IL-1α may reduce chronic inflammation and limit angiogenesis and subsequent prostate tumorigenesis.

## Conclusions

The data presented here demonstrate for the first time that genetically initiated but non-tumorigenic human prostatic epithelial cells can undergo a permanent tumorigenic process as a result of their exposure to tumor-associated myeloid cells. Our studies indicate that IL-1α promotes the transition from indolent to progressing disease and therefore provides a potential new therapeutic target for treatment of prostate cancer.

### Electronic supplementary material

Below is the link to the electronic supplementary material.

Supplementary material 1 (PDF 397 KB)

## Abbreviations

BPH-TW: BPH-transwell
CAF: Cancer-associated fibroblasts
DC: Dendritic cell
EMT: Epithelial to mesenchymal transition
gDNA: Genomic DNA
PIA: Proliferative inflammatory atrophy
PIN: Prostatic intraepithelial neoplasia
PMN-MDSC: Polymorphonuclear phenotype-myeloid derived suppressor cells
M-MDSC: Monocytic-myeloid derived suppressor cells
qPCR: Real-time PCR
RPCI: Roswell Park Cancer Institute
shRNA: Short hairpin RNA
TAMC: Tumor-associated myeloid cells
