# Supplementary material for: Tumor-associated myeloid cells promote tumorigenesis of non-tumorigenic human and murine prostatic epithelial cell lines
Source: Cancer Immunol Immunother. 2018 Mar 3;67(6):873–83. doi: 10.1007/s00262-018-2143-y (PMC5951898; doi:10.1007/s00262-018-2143-y)
Supplement: Supplementary file 1 — Supplementary material 1 (PDF 397 KB) [file 262_2018_2143_MOESM1_ESM.pdf]

Supplementary Table 1 Definition of myeloid cell populations. The surface markers used to define myeloid cell subsets by flow cytometry

Myeloid Subset	Surface Markers Used
PMN-MDSC	$CD45^{+}CD11b^{+}Ly6G^{hi}Ly6C^{lo/-}$
M-MDSC	$CD45^{+}CD11b^{+}Ly6G^{lo/-}Ly6C^{hi}$
Macrophage	$CD45^{+}CD11b^{+}Ly6G^{lo/-}Ly6C^{hi}F4/80^{+}$
cDC	$CD45^{+}CD11c^{+}$

Supplementary Table 2 qRT-PCR Primer Sequences. The forward and reverse primer sequences are listed. The SV40 large T antigen primers were used for PCR of genomic DNA

Species	Gene	5'-primer	3'-primer
Human	CXCL1	AGGCAGGGGAATGTATGTGCACATCTG	CATGAGAAATGTTGACCACACACTGTG
Human	CXCL5	GTGTTGAGAGAGCTGCGTTG	CTATGGCGAACACTTGCAGA
Human	CXCL8	ACTGAGAGTGATTGAGAGTGGAC	AACCCTCTGCACCCAGTTTTC
Human	IL-1α	GAATGACGCCCTCAATCAAAGT	TCATCTTGGGCAGTCACATACA
Human	IL-1β	AAATACCTGTGGCCTTGGGC	TTTGGGATCTACACTCTCCAGCT
Human	GAPDH	ACCAGCCCCAGCAAGAGCACAAG	TTCAAGGGGTCTACATGGCAACTG
Human	Vimentin	GATGCGTGAGATGGAAGAGA	GGCCATGTTAACATTGAGCA
	SV40 large T antigen	GCTTTGCAAAGATGGATAAAG	ACTAAACACAGCATGACTC

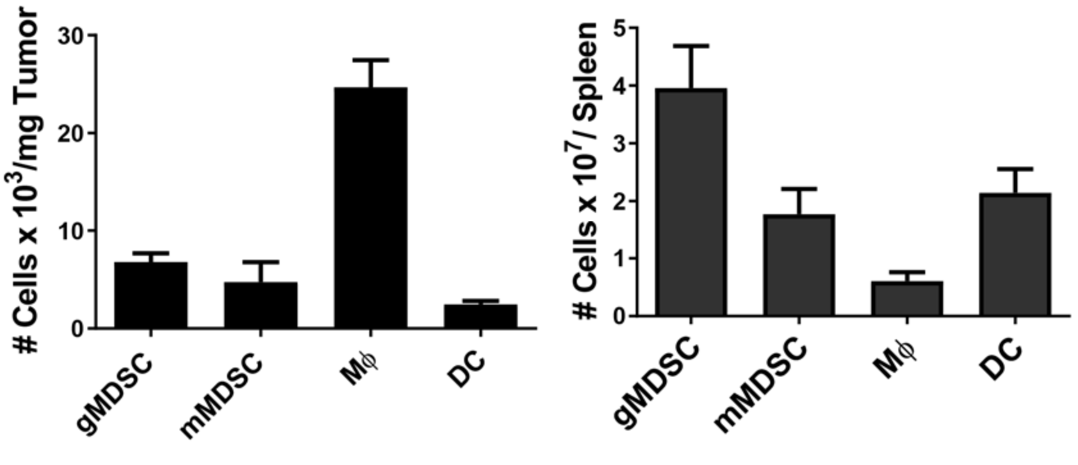

Supplementary Fig. 1 Myeloid cell subpopulations in TRAMP C2 tumor bearing mice. C2 tumors and spleens from tumor bearing mice were harvested and weighed. Flow cytometry was performed to characterize the myeloid cell infiltrate. Results are presented as the mean of four experiments ± SEM.

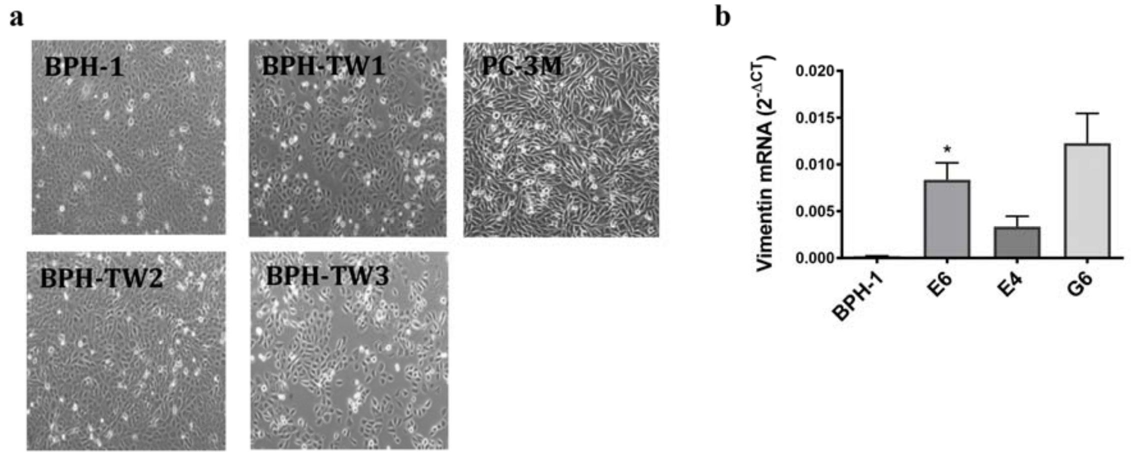

Supplementary Fig. 2 Characterization of BPH-TW cell lines. **a** Light microscopy (20x) of BPH-1, BPH-TW cell lines 1-3 and PC-3M cells. **b** Vimentin mRNA expression. Results are presented as the mean of three experiments ± SEM; *: $P < 0.05$ when compared to expression in BPH-1 cells.

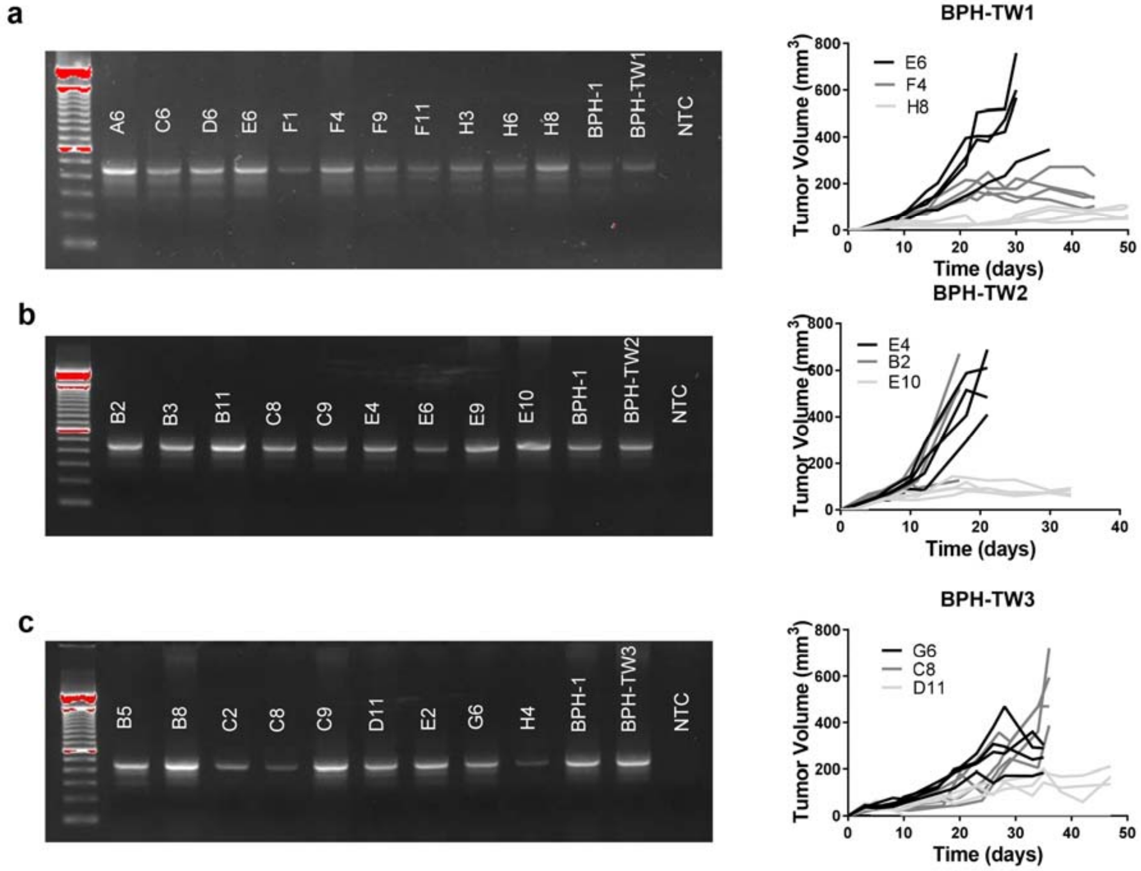

Supplementary Fig. 3 Generation of BPH-TW single cell clones. **a-c Left** SV40 Large T antigen gDNA presence. **Right** Tumor growth kinetics in SCID mice. Each line represents tumor growth in an individual mouse ($n = 4$ for each clone).

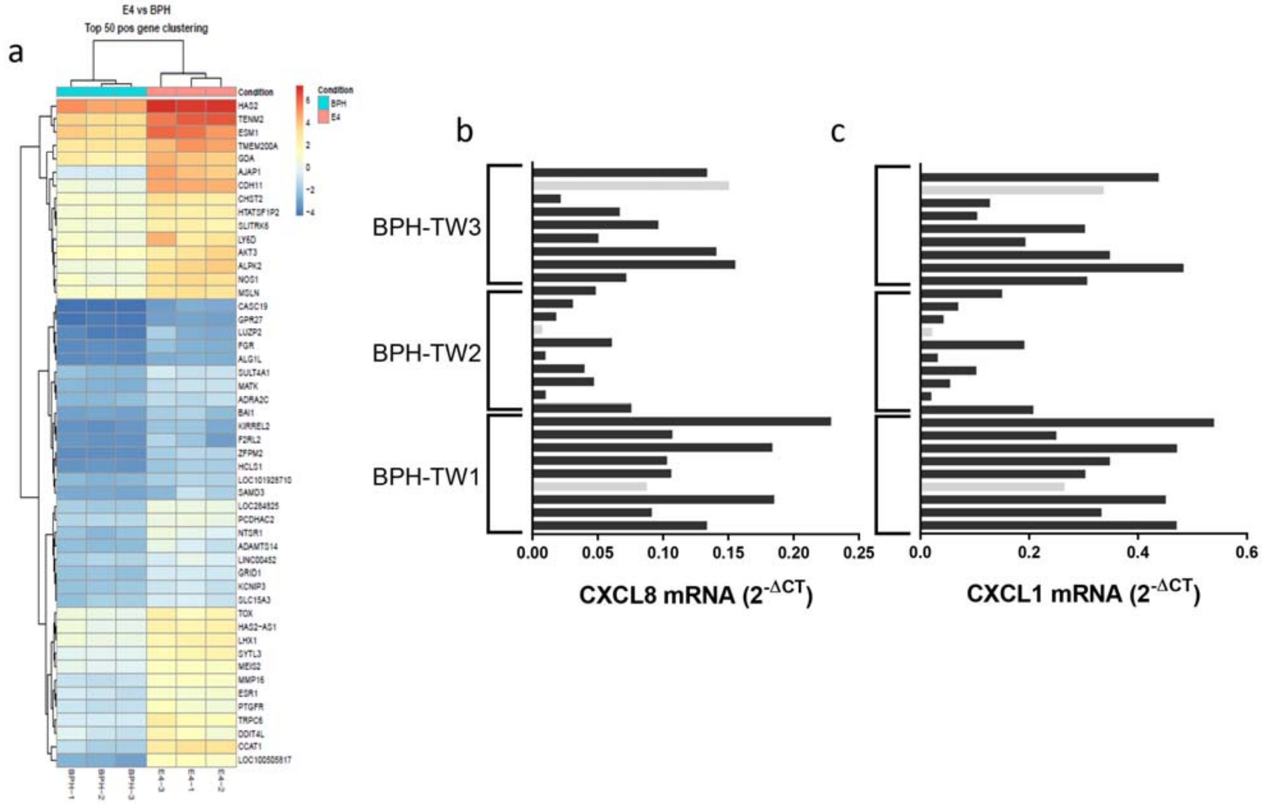

Supplementary Fig. 4 Expression of IL-1 pathway genes in BPH-TW cell lines. **a** Clustergram comparing parental non-tumorigenic BPH-1 RNA to tumorigenic BPH-TW2 clone E4. **b-c** Expression of CXCL8 and CXCL1 mRNA in BPH-TW single cell clones. Selected clones (E6, E4 and G6) are shown in grey in each of the panels.

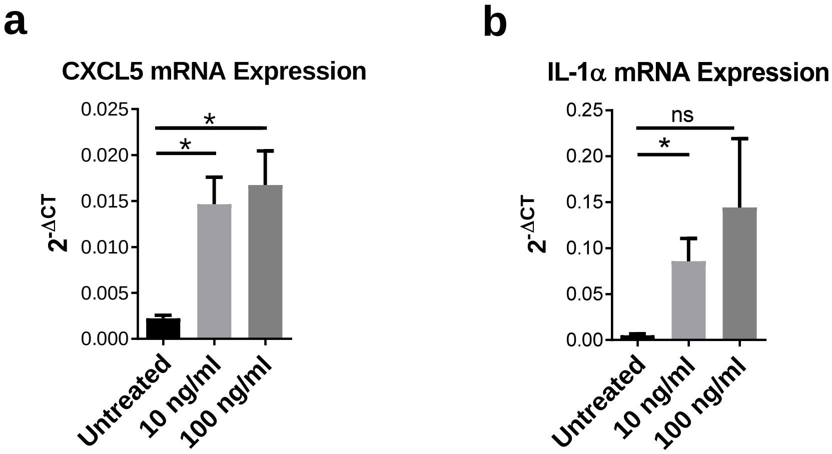

Supplementary Fig. 5 rhIL-1α treatment increases IL-1 gene expression.
a-b BPH-1 cells were treated with recombinant human IL-1α at doses of 10 ng/ml and 100 ng/ml for five days. RNA was isolated from the harvested cells and qRT-PCR was performed for expression of CXCL5 and IL-1α. Results are presented as the mean of four experiments ± SEM. Significance was evaluated using Student *t* test; *:$P < 0.04$ when compared to untreated cells.

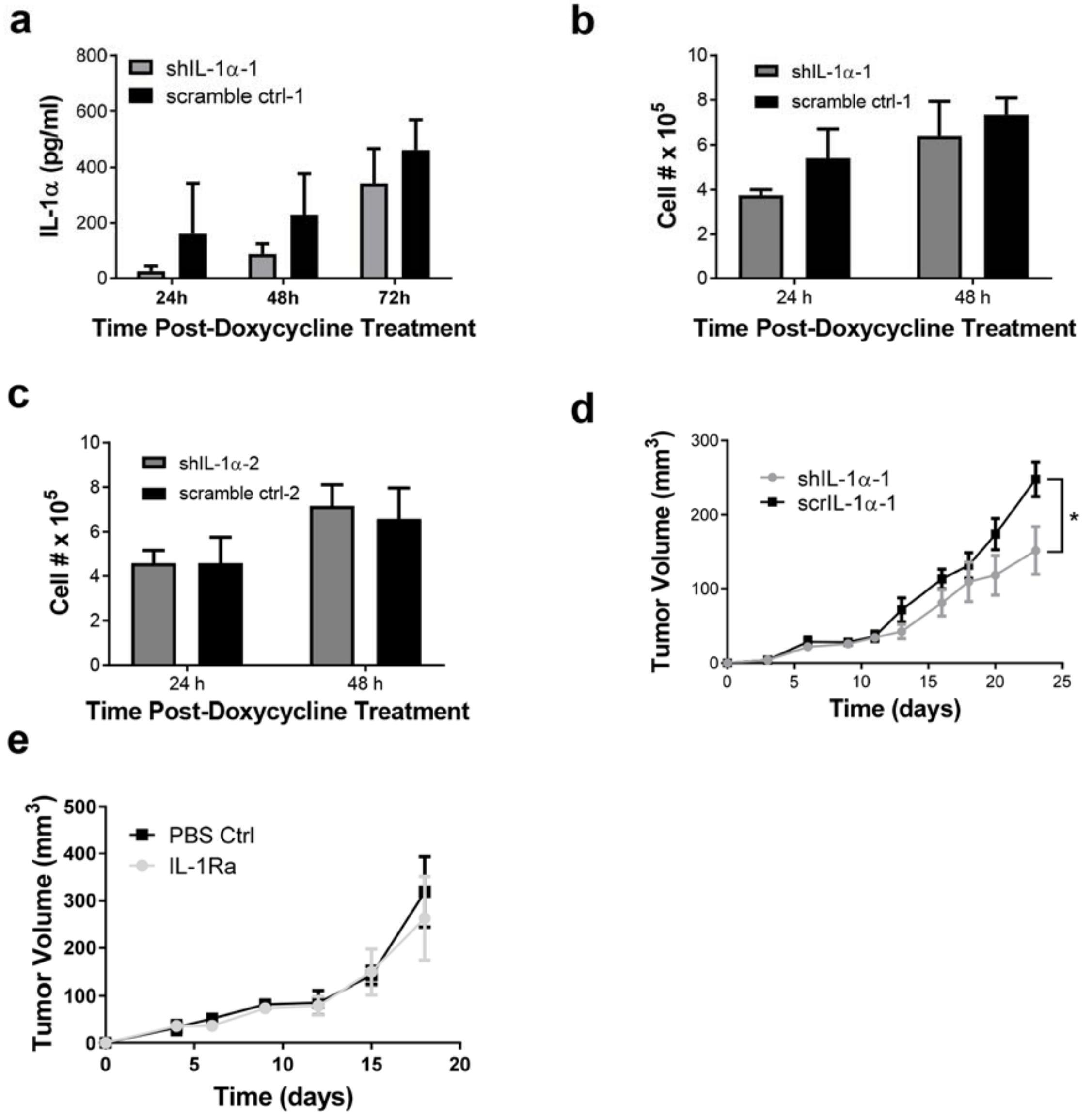

Supplementary Fig. 6 IL-1α knockdown alters tumor growth in E6 cells.
a Doxycycline-inducible E6 shIL-1α and E6 scrIL-1α cells were treated with 1 μg/ml doxycycline for 24-72 hours. IL-1α secretion was tested by ELISA. Results are presented as the mean of three experiments ± SEM. **b-c** Viability of E6 shIL-1α and E6 scrIL-1α cells post-treatment with 1 μg/ml doxycycline. **d** Tumor growth kinetics in doxycycline treated SCID mice injected with E6 shIL-1α or E6 scrIL-1α cells ($n = 4$ per group); $P = 0.0025$. **e** Tumor growth kinetics in SCID mice injected with E4 cells suspended in either PBS or 2.5 μg IL-1Ra ($n = 6$ per group). Every 72 hours, the control group was injected intraperitoneally with 100 μl PBS and the treatment group with 2.5 μg IL-1Ra.
